# Measles Virus Strain Diversity, Nigeria and Democratic Republic of the Congo

**DOI:** 10.3201/eid1611.100777

**Published:** 2010-11

**Authors:** Jacques R. Kremer, Edith Nkwembe, Akeeb O. Bola Oyefolu, Sheilagh B. Smit, Elisabeth Pukuta, Sunday A. Omilabu, Festus D. Adu, Jean-Jacques Muyembe Tamfum, Claude P. Muller

**Affiliations:** Author affiliations: Centre de Recherche Publique–Santé/Laboratoire National de Santé, Luxembourg, Luxembourg (J.R. Kremer, C.P. Muller); Institut National de Recherche Biomédicale, Kinshasa, Democratic Republic of the Congo (E. Nkwembe, E. Pukuta, J.-J.M. Tamfum); Lagos State University, Lagos, Nigeria (A.O.B. Oyefolu); National Institute for Communicable Diseases, Johannesburg, South Africa (S.B. Smit); University of Lagos, Lagos (S.A. Omilabu); and University of Ibadan, Ibadan, Nigeria (F.D. Adu)

**Keywords:** Viruses, measles, epidemiology, Nigeria, Democratic Republic of the Congo, vaccination, genetics, research

## Abstract

We investigated the genetic diversity of measles virus (MV) in Nigeria (2004–2005) and the Democratic Republic of the Congo (DRC) (2002–2006). Genotype B3 strains circulating in Kinshasa, DRC, in 2002–2003 were fully replaced by genotype B2 in 2004 at the end of the second Congo war. In Nigeria (2004–2005), two genetic clusters of genotype B3, both of which were most closely related to 1 variant from 1998, were identified. Longitudinal analysis of MV strain diversity in Nigeria suggested that only a few of the previously described 1997–1998 variants had continued to circulate, but this finding was concomitant with a rapid restoration of genetic diversity, probably caused by low vaccination coverage and high birth rates. In contrast, the relatively low genetic diversity of MV in DRC and the genotype replacement in Kinshasa reflect a notable improvement in local measles control.

## Abstract 2

Differences in epidemiologic patterns are only partially explained by vaccination practices.

Despite >90% reduction in the annual measles mortality rate in the World Health Organization (WHO) African Region during 2000–2006 (*1*), measles remains a major cause of deaths in children in sub-Saharan Africa (*2**,**3*). During this period, routine coverage of measles-containing vaccines increased from 56% to 73% in this region, and >200 million children were vaccinated through supplementary immunization activities (SIAs) by December 2004 (*4*). In the Democratic Republic of the Congo (DRC), vaccination coverage with a first dose of measles vaccine increased from 46% in 2000 to 70% in 2005, according to official country reports (*5*). The first major catch-up campaigns were conducted in several provinces in 2002 (Kasaï Oriental, Nord Kivu) and 2004 (Kasaï Occidental, Maniema, Katanga, Maniema, Sud-Kivu) (*6*). In Nigeria, no SIAs took place until 2005, and routine vaccination coverage was persistently low (<40%), at least until 2006 (*3**,**5**,**7*).

Molecular epidemiology has proven to be a major component of measles surveillance because it enables the effect of accelerated measles control activities to be assessed and the elimination of endemic virus strains to be documented. In Africa, indigenous measles virus (MV) genotypes seem to have a distinct geographic distribution (*8**,**9*). In the central and western parts of sub-Saharan Africa, mainly clade B viruses have been identified (*10**–**17*). The most common genotype is B3, with its 2 clusters B3.1 and B3.2 (*10*). The first B3 sequences in Africa were reported from Nigeria (1997–1998). The 41 MV isolates collected in southwestern Nigeria clustered in 2 distinct subgroups of genotype B3 (B3.1 and B3.2), with an unprecedented maximal sequence diversity of 4.6% in the C-terminus of the MV nucleoprotein hypervariable region (MVN-HVR) (*10*).

In the eastern and southern parts of Africa, genotypes D2 and D4 dominated and a new genotype (D10) was detected in Uganda in 2000 (*8**,**9**,**18*). Although MV sequence data from Africa have been greatly expanded since characterization of the first endemic strains was reported (*8**,**10**,**17**,**19*), essential genetic baseline information is still missing from many countries (*20*). For instance, from DRC, only 5 genotype B3 sequences have been reported from Kinshasa (2000) (*15*). We characterized MV strains collected during 2002 through 2006 from different locations throughout DRC and Nigeria. A comparison of the genetic diversity of MV strains showed notable differences in epidemiologic patterns in both countries that can be only partially explained by differences in vaccination practices.

## Materials and Methods

### Clinical Specimens and RNA Extraction

Clinical specimens from 84 patients with suspected measles were collected in different healthcare centers of the National Measles Surveillance Network in DRC during 2002–2006. Samples from Kinshasa (n = 53) were collected in 14 of the 35 local health districts. Additional specimens were obtained from 5 other provinces of DRC: Bas-Congo (n = 15), Kasaï Oriental (n = 6), Nord-Kivu (n = 2), Sud-Kivu (n = 3), and Maniema (n = 5).

In Nigeria, clinical specimens were collected from patients with suspected measles in Oyo (n = 16), Lagos (n = 12), Adamawa (n = 17), Borno (n = 1), and Sokoto (n = 4) states during 2004 and 2005. Most samples were from hospitalized patients, but those from Adamawa State were obtained during home visits.

Clinical sample collection and MV isolation on VeroSLAM cells were performed as recommended by WHO (*21*). Specimens used for RNA extraction included throat swabs (n = 75), oral fluid (n = 19), MV culture supernatant (n = 19), serum (n = 12), dried blood (n = 5), urine (n = 2), and peripheral blood leukocytes (n = 2). Total RNA was extracted from 140 µL of body fluids, eluted swab specimens, or virus culture supernatant by using the QIAamp Viral RNA Kit (QIAGEN, Hilden, Germany). Most measles cases were also serologically confirmed by measles-specific immunoglobulin M detection by using a commercial ELISA (Enzygnost anti-Measles IgM; Dade-Behring, Marburg, Germany).

### Reverse Transcription–PCR and Sequencing

Specific cDNA of MV nucleoprotein was synthesized by reverse transcription by using SuperscriptIII Reverse Transcriptase (Invitrogen, Merelbeke, Belgium) and random hexamers (Invitrogen). MV cDNA was amplified by nested PCR by using primers MN5 (nt 1113–1134, 5′-GCCATGGGAGTAGGAGTGGAAC-3′ [*22*]) and MN6 (nt 1773–1754, 5′-CTGGCGGCTGTGTGGACCTG-3′ [*22*]) for the first round and primers Nf1a (nt 1199–1224, 5′-CGGGCAAGAGATGGTAAGGAGGTCAG-3′) and Nr7a (nt 1725–1703, 5′-AGGGTAGGCGGATGTTGTTCTGG-3′) for the second round. Both PCRs were performed in a total volume of 25 μL that contained 1.8 mmol/L MgCl_2_, 1× PCR buffer, 0.2 mmol/L dNTPs, 0.5 U Platinum Taq (Invitrogen), and 0.8 μmol/L forward and reverse primer (Eurogentec, Seraing, Belgium). One microliter of cDNA or 5 μL of first-round product (diluted 50× in water) was added as template. Cycling conditions were initial denaturation at 94°C for 2 min; 35 (first round) or 30 (second round) cycles of amplification at 94°C for 30 s, 55°C (first round) or 58°C second round) for 1 min, and 72°C for 1 min; and a final extension at 72°C for 5 min.

Nested PCR products were purified by using the Jetquick PCR product Purification Spin Kit (Genomed, Lohne, Germany). Twenty-five cycles of cycle sequencing (2-min elongation) were performed by using a BigDye Terminator version 3.1 Cycle Sequencing kit (Applied Biosystems, Nieuwerkerk, the Netherlands) with Nf1a or Nr7a primers (0.5 μmol/L) and 10 ng of purified PCR product. Cycle sequencing products were analyzed on an ABI 3130 Genetic Analyzer (Applied Biosystems). Sequences were aligned by using ClustalW (*23*), and phylogenetic trees were constructed by using the neighbor-joining method (Kimura 2-parameter) and MEGA4 software (*24*). All new sequences were submitted to GenBank under accession nos. FN985102–FN985162.

## Results

### Kinshasa, DRC, 2002–2003

**Figure 1:**
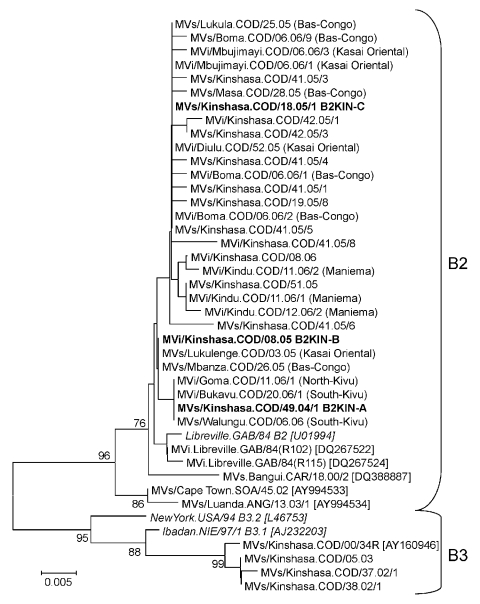
Phylogenetic tree including genotype B2 and genotype B3 of measles virus (MV) strains from the Democratic Republic of the Congo 2000–2006, and World Health Organization (WHO) reference strains (*italics*) of the corresponding genotypes and some other genotype B2 strains available in GenBank (accession numbers in brackets). MV strains were named according to WHO nomenclature: MVi/City of isolation.Country/epidemiologic week.year of isolation/isolate number. Sequences obtained from RNA extracted from isolates (MVi) or clinical material (MVs) were distinguished. The main genotype B2 variants from Kinshasa (B2KIN-A, B2KIN-B, and B2KIN-C) are indicated in **boldface**. Except for B2 strains from Kinshasa, the provinces of Democratic Republic of the Congo where strains were collected are indicated in brackets. The phylogenetic tree was calculated on the basis of the 450-nt region that codes for the C-terminus of the MV N protein by using MEGA4 software (*24*) and the neighbor-joining method (Kimura 2-parameter, 1,000 bootstraps). Scale bar indicates nucleotide substitutions per site.

Eight MV strains collected in Kinshasa during September 2002–January 2003 were assigned to genotype B3.1 on the basis of their MVN-HVR sequences (Figure 1). These strains were obtained at the peak of a large epidemic that occurred in Kinshasa during January 2002–December 2003 (*25*). Their sequences differed by only 1 or 2 nt from earlier genotype B3.1 variants found in Kinshasa and Brazzaville, Congo, in 2000 (*15*), which suggests that MV continuously circulated in Kinshasa during 2000–2003, and that the overall genetic diversity of strains throughout this outbreak was relatively low (0.4% in the MVN-HVR).

### Kinshasa, 2004–2006

**Figure 2:**
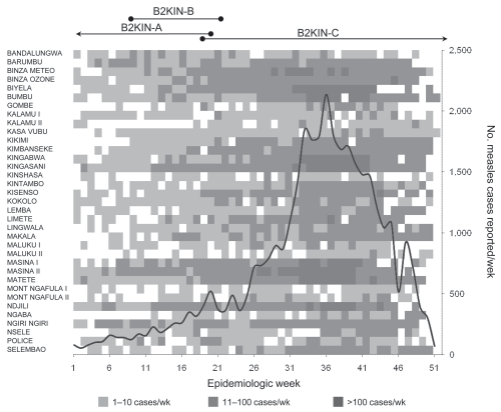
Epidemic curve of measles epidemic in Kinshasa, Democratic Republic of the Congo, 2005. Numbers of reported measles cases per week are shown by epidemiologic week, and measles incidence per week in the 35 health districts of Kinshasa is illustrated by gray shading. The periods during which the main genotype B2 variants (B2KIN-A, -B, and -C) were identified in Kinshasa are indicated above the epidemic curve.

In 2005, another large measles epidemic with >36,000 reported cases and >400 deaths occurred in Kinshasa. Increasing numbers of cases were reported from the last quarter of 2004 until the epidemic peak was reached in epidemiologic week 36 in 2005 (Figure 2). Thereafter, the incidence steadily decreased and fewer cases were reported in the beginning of 2006. Variable numbers of cases were reported from the 35 health districts throughout 2005 (Figure 2), but the case distribution over time suggested 1 large epidemic with a variable effect in the different health districts in the capital of DRC rather than a series of smaller epidemics, as was suggested for the outbreak in 2002–2003 (*25*).

MVN-HVR sequences were obtained from 45 strains collected in 14 of the 35 health districts of Kinshasa during December 2004–February 2006. All 45 viruses belonged to genotype B2, and essentially 3 sequence variants (B2KIN-A, B2KIN-B, and B2KIN-C; Figure 1) were identified. Eleven viruses collected during December 2004–May 2005 had identical sequences in the MVN-HVR (variant B2KIN-A). Five strains collected during February–May 2005 corresponded to the second variant (B2KIN-B), differing by only 1 nt from B2KIN-A in the same gene segment. The third sequence variant (B2KIN-C) was first detected in May 2005 and continued to circulate at least until January 2006. This variant differed by 1 and 2 nts from B2KIN-B and B2KIN-A, respectively, and was identified in 18 strains collected during this period. Eleven other sequence variants were obtained, each from 1 patient, and all but 1 were collected after the peak of the epidemic. Their sequences differed by 1 to 3 nts from B2KIN-C and by 2–5 nts from B2KIN-A and B2KIN-B (Figure 1). Thus, 2 different MV genotypes, B3 and B2, were associated with 2 consecutive measles epidemics in Kinshasa, suggesting that MV circulation had been temporarily interrupted during the intraepidemic phase in 2004.

### Other DRC Provinces, 2005–2006

Genotype B2 strains detected during 2005 and 2006 in Kasaï Oriental, Bas-Congo, and Maniema provinces were most closely related to those strains identified during the same period in Kinshasa (Figure 1). MV cases from these regions thus seemed to be epidemiologically linked to the epidemic in Kinshasa. All 5 sequences obtained from North and South Kivu during February–May 2006 were identical to B2KIN-A, which was last identified ≈8 months earlier in Kinshasa. In the absence of any information on measles incidence in North and South Kivu during 2004–2006, one may speculate that B2-KIN-A strains could have been imported from the east into Kinshasa in 2004 but continued to circulate at the same time in this region bordering Rwanda. Alternatively, B2-KIN-A strains from Kinshasa might have been introduced into the region around Lake Kivu at an early stage of the epidemic because they were not detected after May 2005 in Kinshasa.

### Nigeria, 2004–2005

**Figure 3:**
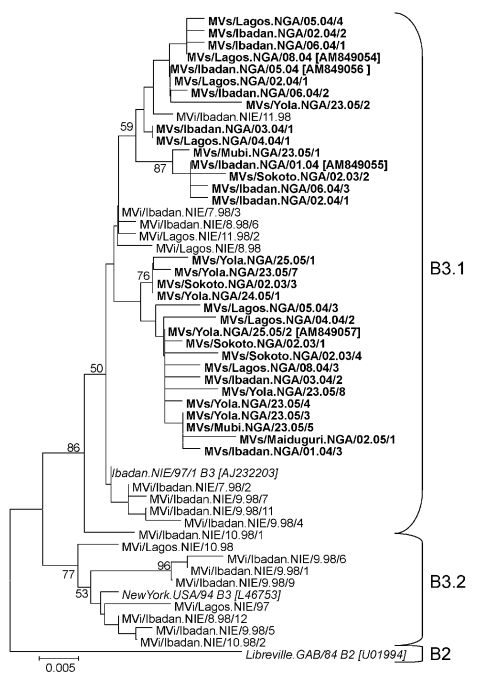
Phylogenetic tree including genotype B3 strains of measles virus (MV) from Nigeria collected in 1997–1998 and 2003–2005 (**boldface**) and World Health Organization (WHO) reference strains of genotypes B3.1, B3.2, and B2 (*italics*). Measles strains were named as indicated in the legend to Figure 1. For all strains from 2003–2005, which have been published, the GenBank accession number is given in brackets. For all strains from 1997–1998, NIE had been used as a 3-letter code for the country (*10*). For the more recent strains, the official WHO 3-letter code NGA was used. The phylogenetic tree was calculated on the basis of the 450-nt region that codes for the C-terminus of the MV N protein by using MEGA4 software (*24*) and the neighbor-joining method (Kimura 2-parameter, 1,000 bootstraps). Scale bar indicates nucleotide substitutions per site.

The MVN-HVR sequence was obtained from 58 viruses collected in 5 states in Nigeria (Oyo, Lagos, Adamawa, Borno, Sokoto) during 2005 and 2006. All sequences were identified as genotype B3 and grouped in 2 separate clusters (Figure 3) with a minimal genetic distance of 5 nts (1.1%) between clusters 1 and 2. Sequence variants of cluster 1 were mainly (81.3%) found in the southwestern states (Oyo, Lagos), whereas most cluster 2 strains (70.6%) were from northern and northeastern states (Adamawa, Borno, Sokoto). Compared with MV sequences obtained in Nigeria during 1997–1998 (*10*), both clusters were most closely related to 1 particular strain (MVi/Ibadan.NIE/7.98/3).

The maximal sequence diversity identified in Lagos, Ibadan, Yola, and Mubi ranged from 1.8% to 2.9%, despite collection of specimens in each city within a period of <2 months. Seventeen sequence variants, differing by 1–11 nts (0.22 to 2.4%) from each other, were distinguished among 28 MV strains collected in January and February 2004 in Lagos (2.2% diversity; Lagos State) and Ibadan (2.2% diversity; Oyo State) (Figure 3).

Ten sequence variants differing by 1–13 nts (0.2%–2.9%) were identified among 17 strains collected in the northeastern Nigeria (Adamawa State) in June 2005. Eight of these sequence variants were detected in 1 city (Yola) during a period of only 2 weeks (2.9% diversity). Another cluster 2 strain (MVs/Maiduguri.NIE/02.05/1), which had a minimum of a 3-nt difference with strains from Adamawa State, was identified a few months earlier in neighboring Borno State. Finally, 4 additional sequence variants (2.7% diversity) were obtained during January 2005 in Sokoto State in northwestern Nigeria. Thus, multiple lineages of genotype B3 were identified in Nigeria, similar to the situation in the late 1990s, indicating that measles continued to be highly endemic during the study period.

## Discussion

This study compared the genetic diversity of MV in 2 countries in Africa of similar size during a similar period. In Nigeria, all viruses belonged to the same genotype (B3.1) and were most closely related to viruses found in the same country in 1997–1998 (Figure 3) (*10*), which suggests an uninterrupted endemic transmission of MV in Nigeria during 1997–2005. MV strains from 2004–2005 formed 2 clusters within subgroup B3.1, both of which were most closely related to 1 particular variant identified in Nigeria in 1998 (MVi/Ibadan.NIE/7.98/3). No B3.2 strains or descendants of most other B3.1 variants from 1997–1998 were detected in 2004–2005, even in samples from the same cities (Ibadan, Lagos) (Figure 3). In 1997–1998, we observed a genetic diversity of 3.1% in Lagos and 4.2% in Ibadan. The phylogeny of the 2004–2005 strains from Nigeria suggests that the 2004–2005 diversity was not caused by multiple chains of transmission sustained since 1997–1998, but by only a few chains of transmission paired with a rapid restoration of a high genetic diversity (2.2% in both cities). Epidemiologic bottlenecks that reduce virus diversity include reduction of susceptible persons, low population size and density, and seasonality of the disease (*15**,**26**–**28*), but the factors that promote viral diversity are less well understood. In Nigeria, the population density is high (e.g., ≈24,000 inhabitants/km^2^ in Lagos in 2005) and vaccination coverage was consistently low at ≈40% throughout the study period (*3**,**5**,**7*). Furthermore, measles incidence declined suddenly at the start of the rainy season (J.R. Kremer, pers. comm.), as was reported for other countries in sub-Saharan Africa (*26*).

**Figure 4:**
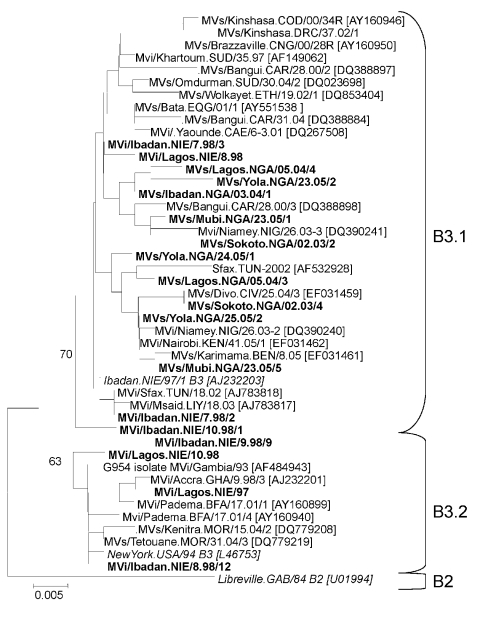
Phylogenetic tree showing a comparison of genotype B3 strains of measles virus (MV) from Nigeria 1997–1998 and 2003–2005 (**boldface)** and representative genotype B3 strains from other countries in Africa available in GenBank (accession numbers in brackets) and World Health Organization (WHO) reference strains of genotypes B3.1, B3.2, and B2 (*italics*). Naming of MV strains and tree calculation were performed on the basis of the 450-nt region that codes for the C-terminus of the MV N protein by using MEGA4 software (*24*) and the neighbor-joining method (Kimura 2-parameter, 1,000 bootstraps). Scale bar indicates nucleotide substitutions per site.

The longitudinal analysis of MV strain diversity in Nigeria confirmed that even in populations in which measles is highly endemic, periods with low measles incidence must occur, during which only a few transmission chains are sustained. The rapid restoration of a high genetic diversity was probably caused by low vaccination coverage, high birth rates and population density, and perhaps MV importation from neighboring countries. Phylogenetic comparison of genotype B3 strains from Nigeria with those from other countries in Africa suggests that transmissions of MV between different countries in Africa are frequent and that the diversity of this genotype in Nigeria reflects the overall genetic diversity of B3 in Africa (Figure 4). In contrast, a noticeably lower genotype B3 strain diversity was found in Sudan during 1997–2000 (1.3% in MVN-HVR) and in Burkina Faso in 2001 (1.5%); these findings were attributed to a higher vaccination coverage or more limited cross-border movement (in the case of Sudan) (*15**,**17*). On the other hand, MV strain diversity in Nigeria was similar to that in the People’s Republic of China (1995–2003), where multiple lineages (5.3% diversity in MVN-HVR) of 1 genotype (H1) co-circulated without obvious geographic restriction (*29*).

In contrast to the situation in Nigeria, where the co-circulation of different B3 viruses continued for years, in Kinshasa, MV circulation was apparently interrupted. The B3 lineage that caused the outbreak in 2002–2003 and that had been detected in 2000 (*15*) in Kinshasa had disappeared in 2004. Genotype B2 strains, which caused another large epidemic in Kinshasa ≈1 year later, seem to have fully replaced genotype B3 strains. It is commonly believed that >95% of a population needs to be immune to interrupt MV transmission (*30*). Because measles vaccination coverage in Kinshasa was suboptimal (<80%, Ministry of Health, DRC) additional epidemiologic constraints must have led to the observed genotype replacement (*31*). The emergence in Germany of genotype D7, which had gradually replaced the indigenous genotypes C2 and D6 during 1999–2001, raised the question of whether genotype D7 strains had a selective advantage with respect to cross-neutralizing antibodies acquired through vaccination or natural infection with other genotypes (*22*).

It has been shown in stochastic models that the likelihood of genotype replacement increases with vaccination coverage and frequency of virus importation, but that it may also occur by chance (*28*). Interestingly, the time point of genotype replacement in Kinshasa coincides with the political reunification of DRC, after the country had been divided into 4 self-governed regions during the second Congo war (1998–2003). The emergence of genotype B2 in Kinshasa may thus have been caused by the massive influx of persons from other provinces where genotype B2 was circulating. Identification of B2KIN-A strains in northeastern DRC (North and South Kivu provinces) in 2006 and detection of B2KIN-B strains in neighboring Rwanda during late 2005 (S. Smit, pers. comm.) is compatible with an importation of genotype B2 from the eastern region of DRC into Kinshasa. On the other hand, B2 strains identified in other provinces seemed to be derived from the epidemic strains in Kinshasa. Epidemics in Bas-Congo and Kasaï Oriental did not start until ≈1 year after the steady increase in measles incidence in Kinshasa, although they were connected to the capital by much frequented roads. Thus, a vaccination campaign during the early outbreak may have prevented many measles cases and deaths not only in Kinshasa, but also in the neighboring provinces, as has been suggested (*32**–**34*).

Genotype B2 was first reported from Gabon in 1984 (*20*) with no similar virus being detected for >15 years. Therefore, genotype B2 was considered inactive, until recent variants of this genotype were found in the Central African Republic (2000), South Africa (2002), and Angola (2003) (*13**,**35*). Viruses obtained from these 3 countries had a minimum genetic distance of 6–8 nts in the MVN-HVR compared with the closest variant (MVi.Libreville.GAB/84[R102]) obtained from Gabon almost 20 years earlier. The B2KIN-B variant differed by only 1 nt from the closest strain identified 20 years earlier in Libreville (Gabon, Figure 1). Thus, several lineages of genotype B2 have probably circulated continuously in central and southern Africa since at least the 1980s, but were never reported because of suboptimal molecular surveillance. Genotype B2 may have circulated in DRC and perhaps even in Kinshasa long before it was first detected in 2004. No MV sequences were available from DRC before this study, except for a few B3 strains from Kinshasa, 2000 (*15*). However, even if we cannot exclude the possibility that genotype B2 was already present during the 2002–2003 epidemic in Kinshasa, B3 seemed to predominate at that time.

## Conclusions

The difference in genetic diversity of MV in DRC and Nigeria is consistent with the level of disease control in both countries during the study period (*5*). In Nigeria, genotype B3 has circulated continuously, at least during 1997–2005. Although transmission of most lineages from 1997–1998 had apparently been interrupted, the genetic diversity observed in 2004–2005 was notable, suggesting that the genetic diversity of MV can rapidly increase in settings with low vaccination coverage and high birth rates.

In DRC, MV circulation has probably decreased because of a notable increase in routine vaccination coverage and SIAs in several provinces. However, the emergence of genotype B2 in 2004–2006 showed that measles incidence can rapidly rise in settings with high birth rates and massive migration of large populations. Of all countries in Africa, DRC and Nigeria reported the largest numbers of measles cases (12,461 and 9,960) in 2008 and the highest average annual measles incidence per 100,000 population during 2005–2008, despite the vaccination of >18 million (DRC) and 60 million children (Nigeria) during recent SIAs (2006–2008) (*7*). Sequence analysis of more recent MV strains from Nigeria and DRC is warranted to evaluate whether these SIAs had any effect on MV strain diversity in both countries.
